# Late Onset Schizophrenia: What Can We Learn? A Qualitative Study of Psychopathology and Social Factors Throughout Life

**DOI:** 10.1192/j.eurpsy.2025.896

**Published:** 2025-08-26

**Authors:** A. Urfer Parnas, C. Krogh, J. Nordgaard, M. Gram Henriksen, J. E. Yttri

**Affiliations:** 1 Psychiatry, Mental Health Hospital of Amager, Copenhagen; 2 Psychiatry, mental Health Center of Roskilde, Roskilde; 3 Psychiatry, mental Health of Slagelse, Slagelse, Denmark

## Abstract

**Introduction:**

In 1943, Manfred Bleuler noted that late-onset schizophrenia (LOS) is an overlooked patient group, but accounts for 20% of all patients diagnosed with schizophrenia. There is a limited interest in this subgroup perhaps because of the focus on patients with early onset of schizophrenia currently. Patients with LOS differ from those with earlier onset by showing better premorbid functioning, cognitive and social skills and a less disorganized. There is limited information about the presence of premorbid near-psychotic or psychotic experiences in these patients.

**Objectives:**

The main aims of this qualitative study were to investigate 1) the extent to which patients with LOS experience near-psychotic or psychotic episodes during their lives prior to their first contact with psychiatry, 2) whether their premorbid social life and level of functioning were affected, and 3) potential triggers before their initial interaction with the mental health care system.

**Methods:**

Inclusion criteria comprised patients with a diagnosis of ICD-10 schizophrenia given at ages 36-59 years. They should not have had any prior contact with the psychiatric healthcare system before their first interaction with psychiatry. A semi-structured interview guide was developed for the study. All interviews were audio recorded and subsequently transcribed. The qualitative analysis consisted of an interaction between theoretical knowledge and empirical results aiming to identify interaction between psychopathology and social factors prior to the first contact with psychiatry.

**Results:**

Most patients with LOS had experienced psychotic symptoms ranging from weeks to years before their first contact with psychiatry. Many had experienced nonspecific symptoms such as depression, fatigue, anxiety or sleeping disturbances for several years before their first psychiatric consultation. The majority reported feeling different throughout their life.

Eleven patients had been married for over three years, six divorced in the months preceding the outbreak. Nine participants had been out of the labor market for several years, the other had been dismissed or went on sick leave shortly just before their contact with psychiatry.

Many stressful life events occurred before their initial psychiatric consultation.

**Conclusions:**

The diagnosis of LOS can easily be overlooked as these patients do not present the typical profile of persons with schizophrenia. However, they experience non specific and psychotic symptoms over years. A closer examination of these patients’ coping strategies during the premorbid phase could provide new psychoeducational insights that may support the recovery of other patients with schizophrenia. The presence of psychotic symptoms several years before the first contact with psychiatry highlights that the onset of the illness is not merely a technical issue, but also a conceptual one.

**Disclosure of Interest:**

None Declared

